# A Review of Carbon Nanofiber Materials for Dendrite-Free Lithium-Metal Anodes

**DOI:** 10.3390/molecules29174096

**Published:** 2024-08-29

**Authors:** Liying Wei, Dawei Ji, Fulai Zhao, Xuwang Tian, Yongshi Guo, Jianhua Yan

**Affiliations:** 1 Key Laboratory of Textile Science & Technology, Ministry of Education, College of Textiles, Donghua University, Shanghai 201620, China; weily1106@163.com (L.W.); guoys02@163.com (Y.G.); 2 College of Chemistry and Chemical Engineering, Shanghai University of Engineering Science, Shanghai 201620, China; 3 Fiber Materials Research Center, School of Textiles and Fashion, Shanghai University of Engineering Science, Shanghai 201620, China; 4 School of Materials Science and Engineering, Shandong University of Technology, Zibo 255000, China; 5 College of Materials Science and Engineering, Key Laboratory of Automobile Materials, Ministry of Education, Jilin University, Changchun 130012, China; txw66688@163.com; 6 Innovation Center for Textile Science and Technology, Donghua University, Shanghai 200051, China

**Keywords:** lithium-metal anodes, lithium dendrites, carbon nanofibers, carbon nanofiber-based composites, lithium-metal batteries

## Abstract

Lithium metal is regarded as ideal anode material due to its high theoretical specific capacity and low electrode potential. However, the uncontrollable growth of lithium dendrites seriously hinders the practical application of lithium-metal batteries (LMBs). Among various strategies, carbon nanofiber materials have shown great potential in stabilizing the lithium-metal anode (LMA) due to their unique functional and structural characteristics. Here, the latest research progress on carbon nanofibers (CNFs) for LMA is systematically reviewed. Firstly, several common preparation techniques for CNFs are summarized. Then, the development prospects, strategies and the latest research progress on CNFs for dendrite-free LMA are emphatically introduced from the perspectives of neat CNFs and CNF-based composites. Finally, the current challenges and prospects of CNFs for stabilizing LMA are summarized and discussed. These discussions and proposed strategies provide new ideas for the development of high-performance LMBs.

## 1. Introduction

With the rapid development of energy storage systems, such as mobile electronic products, laptops, mobile phones and mobile power supplies, it is imperative to urgently develop energy storage technologies with high energy density and safety [1,2]. Lithium-ion batteries mainly based on carbon-based materials have various advantages, such as high operating voltage and no memory effect, and have been widely used in multiple fields [3,4]. However, with the fast growth of new energy vehicles, drones and other high energy storage systems, the demand for energy storage systems with higher energy density and power density has increased. The theoretical energy density of traditional “rocking chair” lithium-ion batteries is less than 400 Wh kg^−1^. Although their energy density is close to its theoretical value, the lithium-ion batteries still cannot meet the growing demand for high energy density in some industries [5,6]. In this context, due to the high theoretical specific capacity (3860 mAh g^−1^), low redox electrochemical potential (−3.04 V vs. standard hydrogen electrode) and low mass density (0.534 g cm^−3^), lithium metal can replace traditional carbon-based materials as the anode to obtain lithium metal batteries (LMBs), such as lithium–sulfur (Li-S) and lithium–oxygen (Li-O_2_) batteries, which can significantly improve the power density and energy density [7,8,9]. For example, when lithium metal is used instead of graphite as the anode and paired with LiCoO_2_ or ternary cathode materials, the energy density can reach 500–1000 Wh kg^−1^. If the cathode is oxygen or sulfur, the battery will be based on multi-electron conversion reactions, and the theoretical energy density can be as high as 3505 Wh kg^−1^ and 2567 Wh kg^−1^, respectively [10,11].

However, while improving the energy density, the LMA also faces severe challenges. The most challenging aspect is the uncontrolled growth of lithium dendrites, mainly due to the uneven distribution of Li^+^ on the entire electrode surface [12]. Due to the “tip effect”, Li^+^ will preferentially accumulate on the fresh lithium surface, resulting in an uneven distribution of Li^+^ flux, leading to the growth of lithium dendrites [13]. The uncontrolled growth of lithium dendrites will further increase the uneven distribution of lithium on the electrode, leading to more severe growth of lithium dendrites. On one hand, the lithium dendrites can accelerate the consumption of electrolyte and lithium, causing the attenuation of active substances and discharge capacity. On the other hand, there is a large amount of “dead lithium” derived from the lithium dendrites, resulting in polarization and low energy density [14,15]. Even, the lithium dendrites can pierce the separator and lead to the internal short circuits of battery [16]. In addition, unlike the anode of lithium-ion batteries, the LMA undergoes the plating/stripping of lithium during the cycling process and cannot serve as the “host” for lithium deposition [17]. Therefore, the deposition behavior of Li^+^ in LMBs belongs to the “no host”, and thus its charging and discharging process is accompanied by infinite volume expansion, which also accelerates the growth of lithium dendrites. In 1988, Canada’s Morley Energy Company went bankrupt due to a fire in LMBs caused by the problem of lithium dendrites [18]. Therefore, achieving a stable dendrite-free LMA is crucial for high-performance and safe LMBs.

**Figure 1:**
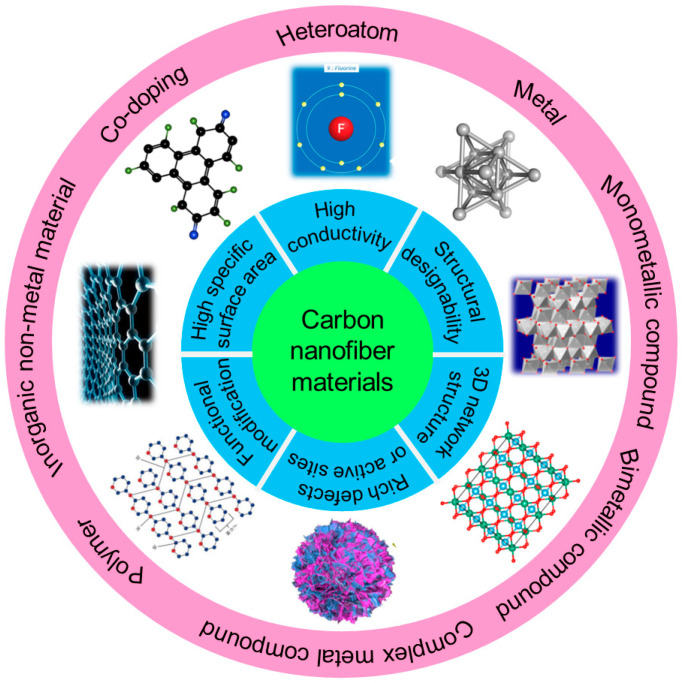
The overview of contents that are summarized and prospected in this review.

At present, there are various methods dedicated to solving the above-mentioned problems, including applying solid electrolyte interphase (SEI) layer protection [19,20,21], homogenizing Li^+^ flux [22,23,24], constructing stable lithium deposition bodies [25,26,27] and implementing electrostatic shielding protection [28,29,30]. In recent years, carbon materials such as graphene, carbon nanotubes (CNTs), carbon nanofibers (CNFs), porous carbon, etc. have been widely explored due to their excellent conductivity, large surface area and chemical stability [31,32]. Among them, CNFs are a one-dimensional (1D) carbon material with nanostructures, a large specific surface area, high electronic conductivity and light weight and has shown enormous potential for large-scale applications in many fields, such as energy storage, nanocomposites, sensors, tissue engineering and biomedical engineering [33,34,35]. One-dimensional CNFs possess many advantages compared to other forms of carbon allotropes (Figure 1). Firstly, a high aspect ratio of several hundred or more can form a conductive network at extremely low penetration, making it suitable for direct use as electrode-active materials and conductive additives [36]. Meanwhile, CNFs possess excellent mechanical strength and flexibility, which are beneficial to the potential application of bendable thin film energy storage devices [37]. More importantly, CNFs can assemble into porous, flexible and three-dimensional (3D) platforms to load active materials and form promising electrode materials [38,39]. At present, there have been various reports about CNFs on implementing stable dendrite-free LMAs, but there are currently no systematic reviews and summaries. This article reviews the advanced protection strategies for LMAs based on carbon nanofiber materials. Firstly, several preparation techniques of CNFs materials are outlined, and then the latest application progress of CNF materials in inhibiting lithium dendrites and protecting lithium anodes are systematically summarized. Finally, the future research directions for CNF materials in LMBs are summarized and prospected.

## 2. Preparation Technology for CNFs

At present, the main methods for preparing CNFs are chemical vapor deposition (CVD), the templated method and electrospinning, and the most commonly used technology is electrospinning. Each of these methods is described in detail in the following sections, and the comparison of various techniques for producing CNF is displayed in Table 1.

### 2.1. CVD

The CVD method originated in 1889 by growing CNFs from carbon-source gases under the action of catalysts [39]. During the CVD process, the gaseous carbon source interacts with the surface of the metal catalyst to form carbon atoms, which then deposit to form nanoscale carbon structures [40]. Firstly, the suitable substrate materials such as silicon substrates or other suitable substrates are prepared, and then the metal catalysts are deposited on the substrate, with the common metals including iron, nickel, cobalt, etc. After pre-treatment, the substrate is placed in a reaction chamber where gases containing carbon sources (methane, ethylene, propylene, etc.) are introduced. These carbon sources interact with the metal catalyst surface and dissociate on its surface to release carbon atoms. Finally, carbon atoms aggregate and diffuse on the surface of the metal catalysts and form the seeds of carbon nanostructures. With the deposition and diffusion of carbon atoms, the carbon nanostructures gradually grow to obtain fibrous structures. During the process, the morphology and structure of CNFs can be controlled by adjusting reaction conditions such as temperature, pressure and gas-flow rate, etc. CNFs produced by CVD are also known as vapor-grown carbon fibers (VGCFs) [37]. Thanks to the high graphitization temperature, VGCFs exhibit excellent conductivity, which can significantly improve the electrochemical performance at low concentrations [41]. However, the high production costs limit the widespread application of the CVD method.

### 2.2. Template Method

The CNF template method is another effective method for preparing CNFs, and anodized aluminum oxide (AAO) is a commonly used template [42]. For example, Pluronic F127 is dissolved in ethanol, then add a resol–ethanol solution containing phenol and formaldehyde. Subsequently, the uniform solution is transferred to an AAO template with an average channel diameter of 25 nm. After ethanol evaporation at room temperature, the AAO template is fully wetted by F127/ressol composite materials. Then, the phenolic resin is thermally polymerized at 100 °C for 24 h, and further carbonized in N_2_ at 700 °C. Finally, CNFs with a diameter close to the template channel diameter are obtained by etching the AAO template with HF [39]. CNFs prepared by template method possess specific pore structures and high specific surface area. Moreover, the structure can be precisely controlled by selecting different templates and precursor materials and adjusting the preparation conditions [43].

### 2.3. Electrospinning

Electrospinning is a fast and efficient strategy to fabricate 1D polymer nanofibers. It utilizes a high-voltage field to stretch polymers or other precursor materials in solutions or melts into nanofibers, possessing the advantages of simple operation, low cost and scalability [44,45]. These polymer nanofibers can serve as the precursors for CNFs and be transformed into CNFs through post-treatment processes such as heat treatment or carbonization. In 1996, Reneker and his colleagues reported a method for preparing CNFs by electrospinning [46]. Firstly, the polymer is dissolved in an appropriate solvent, with various types of commonly used polymers available, including polyacrylonitrile (PAN), polyvinylpyrrolidone (PVP), methyl polyacrylate (PMMA), ethyl polyacrylate (PVA) and polyvinyl alcohol (PEO) [47,48,49,50], etc. Then, the prepared polymer solution is sprayed through a syringe or nozzle, and under a high voltage, the polymer is stretched into extremely fine fibers to form nanoscale fibrous structures. During this process, solvents or volatiles will evaporate and the fiber will gradually cure [51]. Finally, the obtained primary fibers are subjected to heat treatment or carbonization to convert polymer nanofibers into pure CNFs with diameter from 20–1000 nm [52]. During the electrospinning and carbonization processes, the structure and properties of CNFs can be controlled by adjusting the spinning parameters (voltage, solution concentration, receiving distance, etc.) and heat treatment conditions (heating rate, heating program, atmosphere, etc.). Compared with the CVD method, electrospun CNFs possess the advantages of lowest cost, longest length and moderate conductivity. Moreover, some complex structures, such as core-shell, hollow and arranged nanofibers, can also be prepared by adjusting the precursor composition, electrospinning nozzle and post-treatment [53,54], etc. Therefore, electrospinning CNFs have been widely used in Li-S batteries [55,56,57], LMBs [58,59,60], sodium-ion batteries [61,62,63], and other energy storage fields.

## 3. CNFs for Dendrite-Free LMA

As a kind of conductive material, CNF possesses the advantages of a large specific surface area, high conductivity, a 3D network structure, designable structure and abundant defects or active sites, exhibiting enormous potential in inhibiting lithium dendrites growth and protecting lithium anodes. Firstly, CNFs can reduce the local current density of the electrode and balance the charge distribution on anode surface during the charging and discharging process, thus achieving a uniform Li^+^ flux. Secondly, introducing a large surface area 3D network structure can also serve as a host for lithium deposition, helping to slow down volume expansion during the repeated deposition and stripping process. Finally, some lithiophilic materials can be doped into CNFs to regulate the nucleation behavior of lithium and further inhibit the lithium dendrites growth. At present, the CNFs used to stabilize lithium anodes mainly include neat CNFs and CNF-based composites.

### 3.1. Neat CNFs

**Figure 2:**
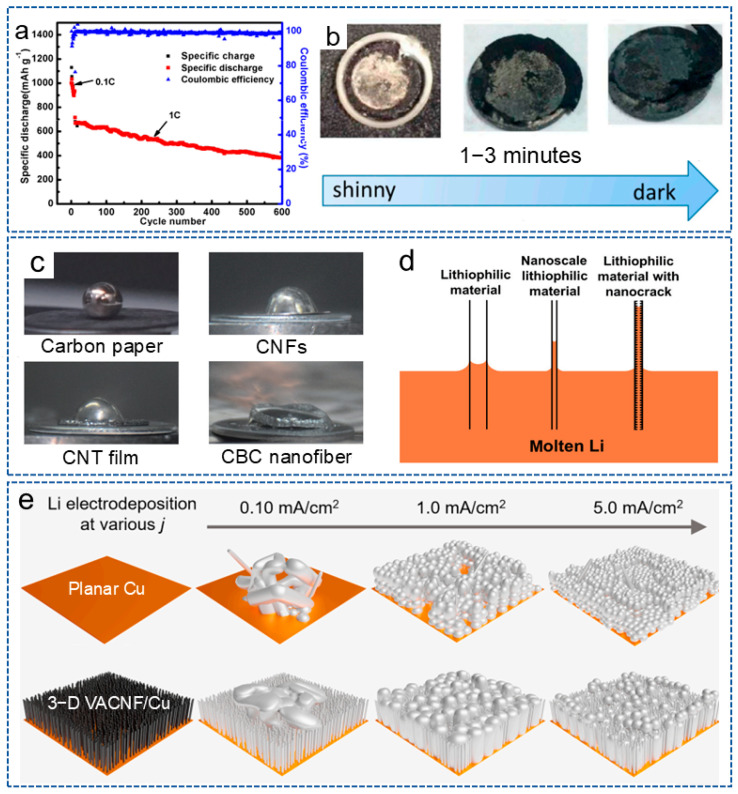
(**a**) The cycling performance of the battery at 1 C. (**b**) The surface of the battery with the anode interlayer after 100 cycles at 0.1 C [64]. Copyright 2017, MDPI. (**c**) The contact angle of different materials. (**d**) Capillary force at different nanoscale materials [65]. Copyright 2023, Springer. (**e**) The effect of plating current density on lithium deposition morphology on planar Cu and VACNF/Cu electrodes, respectively [66]. Copyright 2024, Elsevier.

CNFs possess a large surface area and a high conductive network, which can reduce the local current density of the electrode. In addition, the 3D network structure can also serve as the host for lithium deposition, reducing the volume expansion during the repetitive deposition and stripping processes. For example, Gao et al. [64] used polyimide (PI)-based CNFs as an interlayer for the anode to induce the uniform lithium deposition on the entire electrode surface, thus enhancing the cycling stability of the lithium anode. As shown in Figure 2a, the battery could be stable for 600 cycles, with a coulombic efficiency of 98.8% at 1 C when combined with an N-rich cathode interlayer, and also showed a shiny interlayer surface after cycles (Figure 2b). In recent years, more and more biomass materials have been applied in LMBs. Bacterial cellulose nanofiber is a nanoscale fiber derived from bacterial cellulose with outstanding biocompatibility, biodegradability, renewability, excellent mechanical properties and special surface activity and has also been applied to stabilize lithium anodes. Zhang et al. [65] reported a framework composed of carbonized bacterial cellulose (CBC) nanofibers, which had an inherent lithiophilic property for molten lithium. The surface functional groups and high surface roughness generated by the nano cracks significantly improved the wetting behavior of molten lithium (Figure 2c,d). Thus, the composite anode not only provided a uniform nucleation site for stable lithium stripping/plating, but also adapted to the volume of fluctuations of lithium during the long-term cycling process.

Research has shown that the microstructure of the host also has a significant impact on the lithium deposition behavior. Therefore, Li et al. [66] prepared a vertically aligned CNF (VACNF) array as the model 3D conductive carbon host. As shown in Figure 2e, compared with planar Cu collector, due to the high surface area and lithiophilic properties, the VACNF array provided higher stability and reversibility for lithium plating/stripping. Meanwhile, at a moderate electroplating current density (~1.0 mA/cm^2^), lithium infiltrated the VACNF array more uniformly, resulting in the highest cycling performance of the battery. These results indicate that constructing an ordered 3D nano/microstructure can effectively regulate the nucleation of lithium metal and guide its deposition.

In short, the large surface area and high electrical conductivity of CNFs can reduce the local current density of the electrode, and the 3D network structure can also serve as the “host” of lithium deposition to reduce volume expansion during the cycling process. In addition, a special fiber arrangement or pore structure can be designed to effectively prevent the growth of lithium dendrites through controlling the deposition direction of lithium from the physical level. In recent years, the development of new green carbon source materials has attracted widespread attention from researchers. It is worth noting that pure CNFs have poor lipophilic properties, resulting in the weak interaction with lithium, inevitably leading to a large nucleation barrier. Thus, how to improve the lipophilic properties of CNFs materials has become a key issue for researchers. In addition, the polymer precursor used for preparing flexible carbon nanofiber materials is relatively limited in variety. For example, the precursor of electrospinning flexible CNFs is mostly PAN based on organic solvents, which is not conducive to the environmentally friendly preparation of materials.

### 3.2. CNF-Based Composites

Although CNFs have shown excellent performance in stabilizing lithium anodes, most of them are non-polar materials with weak interactions with Li^+^. Lithiophobic carbon surfaces are not conducive to the nucleation and deposition of lithium, and can only regulate the deposition behavior at the physical level. Therefore, researchers have attempted to introduce lithiophilic materials into carbon substrates to obtain CNF-based composites, including heteroatom, metal, metal compounds, inorganic non-metal material, polymer and co-doping.

#### 3.2.1. Heteroatom/CNF Composites

**Figure 3:**
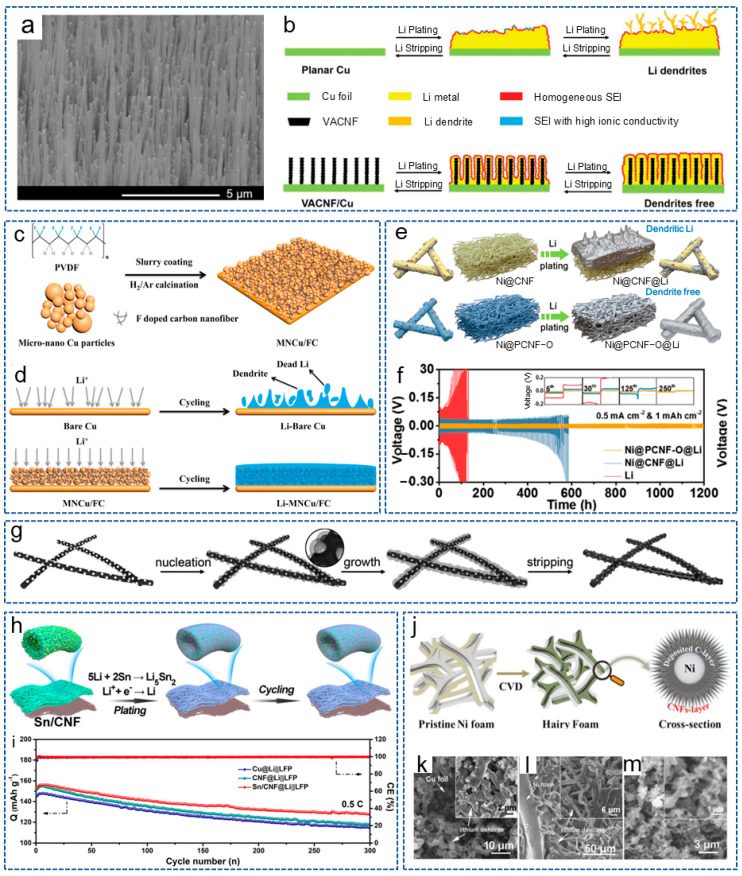
(**a**) Scanning electron microsphere (SEM) image of VACNF array surface. (**b**) Schematic diagram of lithium-cycling process in planar Cu electrode and VACNF/Cu host [70]. Copyright 2020, Wiley-VCH. (**c**) Schematic diagram for the preparation process of MNCu/FC. (**d**) Schematic diagram of lithium plating/stripping process on Cu and MNCu/FC host [71]. Copyright 2020, Elsevier. (**e**) Schematic illustration of lithium plating behavior with Ni@CNF and Ni@PCNF-O. (**f**) Cycling performance of symmetric cells with different electrodes [72]. Copyright 2021, Elsevier. (**g**) Schematics of lithium nucleation/growth process induced by Ag nanoparticles seeds on CNFs [73]. Copyright 2017, Wiley-VCH. (**h**) Schematic illustration of lithium plating/stripping on Sn/CNF. (**i**) Cycling performance of different cells at 0.5 C [74]. Copyright 2023, The Royal Society of Chemistry. (**j**) Synthesis process diagram of CNF@Ni foam. (**k**–**m**) SEM images of (**k**) Cu foil, (**l**) Ni foam and (**m**) CNF@Ni foam after 100 cycles at 1 mA cm^−2^ [75]. Copyright 2020, Wiley-VCH.

As is well known, chemical doping can enhance the energy storage and improve the electrochemical activity of CNFs. Studies have shown that introducing lithiophilic sites on conductive substrates can regulate the deposition behavior of lithium. This is because the abundant lithium sites can uniformly distribute Li^+^ on the substrate surface and prevent high Li^+^ flux on the “hotspot” [67,68]. Zhang et al. [69] prepared a lightweight, free-standing, N-doped CNF 3D matrix (NCNF), with appropriately doped N providing a large number of lithium nucleation sites. Compared with Cu collectors and conventional scaffolds, NCNF effectively avoided the dendrite formation and volume changes. Similarly, Cheng et al. [70] directly grew vertically arranged CNF arrays on a planar Cu collector (VACNF/Cu) as a 3D host with high porosity for the lithium anode. The surface image of the VACNF array is shown in Figure 3a. Figure 3b shows the schematic process of lithium plating/stripping in a planar Cu electrode and the VACNF/Cu host. Excellent conductivity and highly active lithiophilic graphitic edge sites facilitated the uniform lithium plating/stripping around each VACNF, while N-heteroatom doping enabled a lithiophilic carbon surface and facilitated uniform lithium deposition. Therefore, the symmetric cells achieved a low voltage hysteresis of 35 mV over 500 h under 1 mA cm^−2^ and 2 mAh cm^−2^.

In addition to N, F is also a commonly doped atom. Wang et al. [71] obtained a 3D micro-nano-structured Cu skeleton with a crosslinked F-doped CNF network (MNCu/FC) by the slurry-coating method (Figure 3c). As shown in Figure 3d, the conductive 3D structure constructed by the copper skeleton and CNF network greatly accelerated charge transfer and inhibited the of lithium dendrites’ growth. Moreover, the lithiophilic network composed of F-doped CNFs is conducive to the uniform nucleation and deposition of lithium. As a result, the symmetric battery showed a long lifespan of 600 h at 1 mA cm^−2^ and 1 mAh cm^−2^. Recently, Sun et al. [76] introduced single-atom Co-Nx sites into the CNF framework, achieving a transition from lithiophobic to lithiophilic of the carbon framework to reduce the dendrite formation. Thanks to the synergistic effect between the atomically dispersed Co-Nx sites and the 3D conductive network, the Li-S batteries exhibited excellent cycling stability.

#### 3.2.2. Metal/CNFs Composites

Nanoparticles refer to particles at the nanoscale, which possess excellent conductivity or special optical properties, and have been widely used in the fields of biology, materials science and energy storage [77]. In addition, due to their advantages of rich active sites, a high specific surface area, controllable size and volume, nanoparticles are also widely applied in LMBs. It has been reported that metal nanoparticles can act as the “seeds” to induce lithium deposition behavior or form alloys with lithium owing to the high solubility in lithium, and have been used to regulate nucleation behavior. The introduction of nucleation seeds will increase the lipophilic property of the CNF skeleton and induce a large critical nuclei size and a small nucleation overpotential [78]. For example, Wang et al. [79] prepared a CNF mat modified with silicon nanoparticles (Si@CNFs) and used as a novel lithiophilic conductive interlayer. The Si@CNFs interlayer possessed a 3D porous conductive structure, which could act as redistributor and host of lithium to regulate the deposition behavior of lithium. Analogously, Wang et al. [80] synthesized a layered porous CNF network containing single Zn atoms (ZnSA@HPCNF) by electrospinning and carbonization. As an anode host, the composite structure possessed excellent chemical anchoring and lipophilic properties. Thus, the symmetric battery exhibited low overpotential during repeated plating/stripping for 900 h at 5 mA cm^−2^. Ulteriorly, Gao et al. [32] constructed a 3D freestanding CNF modified with lipophilic metal particles (CNF/Me, Me = Sn, Fe, Co) by electrospinning. The introduction of metal particles effectively prevented the formation of lithium dendrites. As expected, the battery based on CNF/Sn-Li composite anode showed a stable cycle of 2350 h at 1 mA cm^−2^ and 1 mAh cm^−2^. To achieve the dendrite-free LMA and stable S cathode at the same time, Liu et al. [72] designed an oxygen-functionalized mesoporous CNF framework decorated with Ni nanoparticles (Ni@PCNF-O). Compared with Ni@CNF, the regulated electric field generated by the oxidized mesoporous structure of Ni@PCNF-O promoted the uniform nucleation and growth of dendrite-free lithium metal (Figure 3e). Meanwhile, the existence of oxygen-containing groups effectively mitigated the shuttle effect. As shown in Figure 3f, the Ni@PCNF-O@Li anode showed a long lifespan of over 1200 h, with an overpotential of 17 mV at 0.5 mA cm^−2^.

As a highly conductive material, the introduction of Ag nanoparticles into the carbon matrix can increase the electroactive specific surface area and lipophilic properties of the materials, which has been widely investigated by researchers. Hu et al. [73] synthesized ultrafine Ag nanoparticles distributed uniformly on CNFs by the rapid Joule heating method. As shown in Figure 3g, Ag nanocrystalline seeds effectively reduced the nucleation overpotential of lithium metal and guided the lithium uniform deposition of lithium on the 3D carbon matrix, effectively solving the lithium dendrite problem of LMA. Yan et al. [59] prepared Ag nanoparticle-doped porous CNFs (Ag-PCNFs) by electro-blown spinning technology and coated them on the side of the commercial separator near the lithium anode. The porous CNFs, with their high specific surface area, improved the lithium-loading capacity. Meanwhile, the Ag nanoparticles contributed to promoting the electrochemical reaction and reducing the local current density. The modified Li-S battery achieved a high capacity retention rate of 77.73% after 600 cycles at 0.5 C.

In addition, alloying reactions are another common strategy for lithium anode protection. Due to the fact that the formed alloys possess similar crystalline structure with lithium and outstanding wettability to lithium, the matrix is more likely to induce lithium deposition [81]. Therefore, by initiating a similar alloying reaction with lithium, Li^+^ are induced to deposit uniformly on the interior or surface of the CNF matrix. Wang et al. [82] added Sn nanoparticles into the PAN spinning solution and obtained a lithiophilic CNF framework coated by a thin layer of Sn after carbonization. The Sn layer endowed the carbon skeleton surface with lipophilic properties and the ability to form alloys. The alloy interlayer provided uniform and high-density nucleation sites to achieve the smooth lithium electrodeposition, therefore suppressing the dendrite growth and mitigating the CNF volume expansion. Inspired by the above research, Liu et al. [74] constructed a self-supporting Sn-modified porous CNF skeleton (Sn/CNF) based on PAN. Figure 3h displays the schematic illustration of lithium plating/stripping on Sn/CNF. During the initial lithiation stage, lithiophilic Li_5_Sn_2_ with a low nucleation overpotential was formed, derived from the electrochemically generated alloying reaction of lithium and Sn. The Li_5_Sn_2_ alloy acted as the continuous lithium nucleation grains and promoted the charge-transfer kinetics to homogenize the Li^+^ flux, and the battery with LiFePO_4_ (LFP) displayed a steady cycle for 300 cycles with a high coulombic efficiency of 99.6% (Figure 3i). In addition to in situ generation of alloys through reacting with lithium, there are also studies that directly doped metal alloys into the CNF frame. Liu et al. [83] prepared a N-doped porous CNF decorated with high lithophilic FeNi alloy nanoparticles. The FeNi alloy and porous structure exposed more nucleation active sites, thus reducing the nucleation overpotential and improving the lithium deposition behavior.

Currently, most metals are introduced into the CNF matrix in the form of nanoparticles. In fact, the form of the metal also affects the electrochemistry of the entire system. Interestingly, Liu et al. [75] grew CNFs in situ on 3D conductive capillary Ni foam to form the CNF@Ni current collector by CVD (Figure 3j). On the one hand, the Ni foam with a hierarchical structure provided sufficient active sites for lithium nucleation. On the other hand, CNFs with protruded tips greatly increased the electrochemically active surface area and reduced the local current density. Thus, the symmetric cells based on CNF@Ni-Li could be stably cycled for 1000 h with low voltage hysteresis, and the Li-S full cell also showed a high capacity of 400 mAh g^−1^ after 800 cycles at 5 C. As shown in Figure 3k–m, compared with Cu foil and Ni foam, there were no significant lithium dendrites of the CNF@Ni foam after 100 cycles at 1 mA cm^−2^.

Constructing a hydrophilic–hydrophobic gradient current collector is an effective strategy to solve the lithium dendrite growth. Based on this, Cao et al. [84] prepared a hybrid CNF structure composed of a gradient N-doped CNF layer and a Cu-doped CNF layer. The gradient doped N offered N-rich sites for lithium deposition, and the CuCNF capping layer maintained a deposit-free surface. More importantly, the CuCNF-NCNF hybrid collector achieved a high coulombic efficiency and resistance to repeated stress application.

#### 3.2.3. Metal Compound/CNFs Composites

In addition to metals, some metal compounds, including metal fluorides, metal oxides, metal sulfides, metal phosphides, metal carbides, etc., as well as bimetallic oxides, also have a good affinity for lithium, which can significantly enhance the lipophilic properties of CNFs. Meanwhile, each metal compound possesses its own advantages, and introducing them into the carbon matrix can combine the induced growth nucleation mechanism with physical barriers to prevent the formation of lithium dendrites and improve the safety of the battery.

Monometallic Compound/CNF Composites
Metal Oxide/CNFs Composites

**Figure 4:**
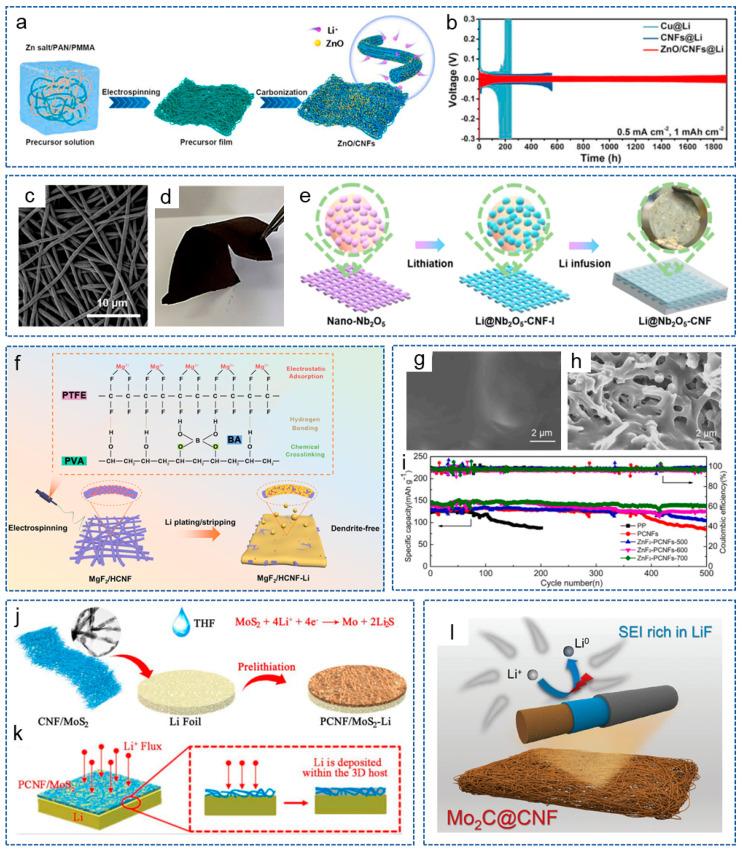
(**a**) Schematic illustration for preparation process of ZnO/CNFs. (**b**) Cycling performance of symmetric battery with different electrodes at 0.5 mA cm^−2^ [85]. Copyright 2021, Elsevier. (**c**) SEM images of Nb_2_O_5_-CNF membrane. (**d**) Optical photograph of flexible Nb_2_O_5_-CNF membrane. (**e**) Schematic illustration of the preparation process for Li@Nb_2_O_5_-CNF [87]. Copyright 2021, American Chemical Society. (**f**) Schematic illustration of the synthesis process for MgF_2_/HCNF and lithium deposition process on the MgF_2_/HCNF-Li electrode [92]. Copyright 2024, American Chemical Society. (**g**) Surface morphologies of lithium anode in symmetric batteries after cycles. (**h**) Surface morphologies of ZnF_2_-PCNFsinterlayer after cycles at 1 mA cm^−2^. (**i**) Cycling performance of different cells at 1 C [94]. Copyright 2021, Elsevier. (**j**) Preparation process diagram for the PCNF/MoS_2_-Li composite anode. (**k**) Schematic diagram of lithium deposition process on PCNF/MoS_2_-Li anode [97]. Copyright 2022, Elsevier. (**l**) Schematic diagrams of Mo_2_C@CNF after lithium deposition [100]. Copyright 2023, Wiley-VCH.

Metal oxide is a kind of common metal compound with lipophilic properties and has become a hot spot in the research on resistance to lithium dendrites in LMBs. Wang et al. [85] prepared ZnO/CNFs composite materials based on electrospinning and formed a composite anode by electroplating lithium (Figure 4a). The interconnected 3D structure provided a rich electrolyte/electrode interface, which helped to reduce the local current density. Meanwhile, the uniformly distributed ZnO grains effectively enhanced the lithiophilic property of CNFs. As shown in Figure 4b, the ZnO/CNFs@Li composite anode showed a long lifespan of 1900 h at 0.5 mA cm^−2^. To achieve a dendrite-free LMA, Fan and his colleagues [86] fabricated a lithiophilic CuO-CNF 3D current collector combining a 3D CNF skeleton with CuO nanoparticles. Firstly, the 3D CNF skeleton reduced the local current density by mitigating the volume changes during repeated plating/stripping processes. Secondly, the lithiophilic CuO brought an ultralow nucleation overpotential, thereby effectively alleviating the growth of lithium dendrites. Furthermore, Long et al. [87] introduced lipophilic Nb_2_O_5_ into CNF and obtained Nb_2_O_5_-CNF as the support for lithium anodes. Figure 4c,d show that the Nb_2_O_5_-CNF membrane possessed abundant porosity and excellent flexibility, respectively. As shown in Figure 4e, CNFs with rich 3D network structures could serve as a host for lithium deposition. When molten lithium was loaded, an in situ lithiation reaction occurred between lithium and the Nb_2_O_5_ nanocrystals to form Li_x_Nb_y_O nanoparticles, thereby regulating the nucleation/growth behavior of lithium. At present, some materials with special structures and channels are designed to guide the lithium deposition direction, which can provide enough ordered space and induce lithium to grow in a reasonable, oriented and ordered direction, similar to a “magnet”. Liu et al. [88] constructed a uniformly parallel multichannel super-hydrophilic ZnO-doped CNF framework (MCCNF@ZnO). This composite skeleton offered abundant super-lithiophilic nucleation sites for stable lithium plating/stripping, thus effectively avoiding “dead lithium” and improving the stability of the battery. The symmetrical battery showed a long sequential performance of 3300 h at 0.5 mA cm^−2^. In order to solve the shuttle effect of polysulfide and the growth of lithium dendrites at the same time, Li et al. [89] designed a dual-function electrode host for the Li-S battery by implanting CNFs with ordered porous Co-decorated Al_2_O_3_ on CNT film (CNTF). Thanks to the 3D structure, CNFs and active Co nanoparticles, the anode could buffer the volume expansion during plating/stripping and achieve the uniform growth of lithium.

Metal Fluoride/CNF Composites

Compared with metal oxides, metal fluorides have received widespread attention from researchers in recent years. Metal fluorides with stronger polarity have an excellent Li^+^ transport ability, and the doping defects of F can effectively adsorb Li^+^ and guide the homogeneous lithium nucleation behavior [90]. Moreover, the electrochemically active C–F bond helps to form a LiF-rich SEI layer, promoting a stable reversible plating/stripping behavior [91]. Ai et al. [92] created a lithium host composed of MgF_2_ nanodots covalently bonded to the honeycomb CNFs (MgF_2_/HCNFs) by an in situ solid-state reaction, solving the problem of the lack of a strong chemical bond between MgF_2_ and carbon. Figure 4f displays the schematic illustration of the synthesis process for MgF_2_/HCNF and the lithium deposition process for the MgF_2_/HCNF-Li electrode. The covalent bond between MgF_2_ and HCNF extended the cycle life of MgF_2_ nanodots. Due to the synergistic effect of lithiophilic MgF_2_ and the capillary force from the unique honeycomb-like CNFs, molten lithium could be quickly impregnated in composite materials. Analogously, Wang et al. [93] introduced AlF_3_ particles into 3D CNFs through electrospinning and prepared an freestanding AlF_3_@CNF multi-functional interlayer, which greatly enhanced the safety and electrochemical performance of the battery. Our group [94] used electro-blow spinning technology to prepare porous CNFs (PCNFs) doped with ZnF_2_ nanoparticles. Then, ZnF_2_-PCNFs were used as functional coating to modify the separator, resulting in a smooth and flat lithium anode morphology (Figure 4g). Moreover, it could be seen from Figure 4h that lithium is uniformly deposited inside or on the surface of ZnF_2_-PCNFs. Thus, the battery could be stable for 500 cycles at 1 C (Figure 4i). By using the similar electro-blow spinning technology, Kang et al. [95] designed YF_3_-doped CNFs based on PAN and acted as the functional layer to modify the commercial separator. The results showed that this functional layer not only inhibited the growth of lithium dendrites, but also effectively suppressed the “shuttle effect” of polysulfides.

Metal Sulfide/CNFs Composites

Metal sulfides are another type of compound used to regulate lithium deposition behavior. Due to the strong ionic bond characteristics, metal fluorides possess strong polarity, which can effectively adsorb Li^+^ and polar polysulfides in Li-S batteries. However, the poor conductivity of metal fluorides is not convenient for electron transportation, and metal sulfides can compensate for this disadvantage [20]. Cheng and his colleagues [96] prepared a CNF flexible interlayer doped with MnS by microfluidic spinning technology, which exhibited excellent electrochemical performance when combined with a lithium anode. On one hand, the flexible carbon film acted as a transitional layer to reduce volume expansion. On the other hand, the doped MnS provided more active sites to guide the homogeneous lithium deposition. Xu et al. [97] designed a CNF framework embedded with edge-enriched and ultra-thin MoS_2_ (PCNF/MoS_2_) to stabilize LMA (Figure 4j). The spontaneous chemical reaction between MoS_2_ and lithium in situ formed Li_2_S and Mo, thereby reducing lithium nucleation overpotential and guiding lithium deposition within the 3D CNF framework, effectively inhibiting the growth of lithium dendrites (Figure 4k). As a result, the PCNF/MoS_2_-Li anode exhibited an excellent long cycle life at a current density of 1 mA cm^−2^.

Other Metal Compound/CNFs Composites

In addition, other metal compounds, including metal phosphide, metal carbides and metal nitride nanoparticles or nanolayers are also applied to regulate the size and location of lithium nucleation. For example, Liu et al. [98] created Ni_2_P-doped flexible CNFs (Ni_2_P@CNF) based on electrospinning and heat treatment, which served as a 3D framework to guide lithium deposition behavior. Recently, Li et al. [99] prepared exposed-edged Cu_3_P faceted nanoparticles anchored along interlaced 3D CNFs (ECP@CNF) by electrospinning and used them as a 3D lithium host. The Cu_3_P nanoparticles with exposed crystal facets provided more lithiophilic sites to guide the orderly lithium deposition. The interlaced CNF with high electrical conductivity prevented charge accumulation. As expected, the ECP@CNF/Li||LFP cells displayed a stable performance for 650 cycles at 1 C. Peng and his colleagues [100] prepared a 3D current collector made of lithiophilic Mo_2_C clusters embedded with CNF (Mo_2_C@CNF). Density functional theory (DFT) proved that Mo_2_C clusters have a lower nucleation overpotential compared to MoO_2_, and the high specific surface area of the 3D current collector contributed to reducing the local current density. Specifically, the transition metal Mo prompted the formation of a LiF-rich SEI layer (Figure 4l), thus eliminating the “dead lithium”. The Li/Mo_2_C@CNF symmetrical cell showed a stable cycle of 600 h, with a low overpotential of 18 mV under 3 mA cm^−2^. 

In recent years, transition metal oxynitrides have demonstrated unique advantages in energy conversion and storage due to their excellent conductivity low bandgap, and they hold great promise for applications in lithium anodes [101,102]. Qiu et al. [103] synthesized a 3D host for LMA through an in situ growing method, wherein CNFs were decorated with uniform CrO_0.78_N_0.48_ nanoparticles (ACrCFs). Owning to the abundant active sites, excellent lithiophilicity and mixed ion-electron conductivity, the modified lithium anodes displayed cyclic stability, with 320 cycles at 1 mA cm^−2^, along with a flat lithium morphology and a low nucleation overpotential of 10.4 mV.

Bimetallic Compound/CNFs Composites

**Figure 5:**
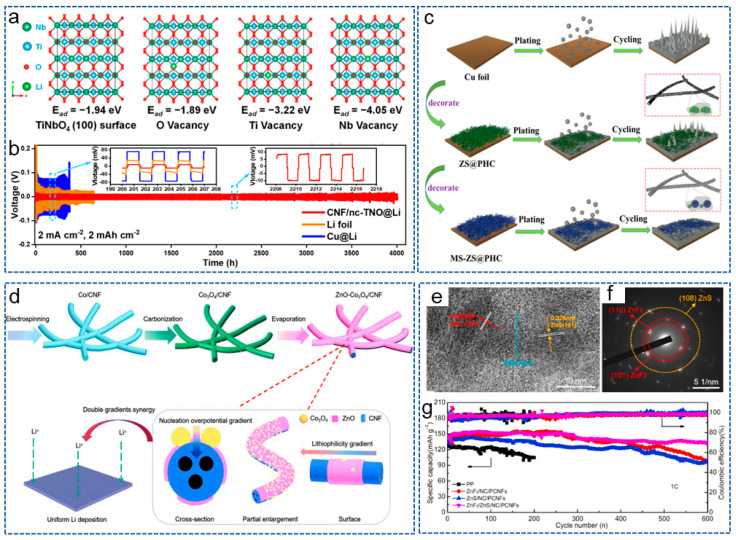
(**a**) DFT adsorption geometry and energy of lithium atoms on TiNbO_4_ (100) surface, including O vacancy, Ti vacancy, Nb vacancy. (**b**) Voltage-time profiles of different symmetrical cells at 2 mA cm^−2^ and 2 mAh cm^−2^ [104]. Copyright 2023, American Chemical Society. (**c**) Schematic illustration of lithium deposition in the different scaffold [106]. Copyright 2022, Elsevier. (**d**) Schematic illustration of synthesis process and working mechanism of ZnO-Co_3_O_4_/CNF [107]. Copyright 2023, Elsevier. (**e**) High-resolution transmision electron microscope image and (**f**) selected area electron diffraction pattern of ZnF_2_/ZnS/NC/PCNFs. (**g**) Cycling performances of Li|LFP batteries with ZnF_2_/ZnS/NC/PCNFs interlayer at 1 C [58]. Copyright 2022, Elsevier.

Bimetallic compounds refer to the compounds composed of two different metal elements, which possess unique physical, chemical and electronic properties, and have attracted widespread attention from researchers in the field of LMBs. Recently, in order to reduce the adverse growth of lithium dendrites, Yang et al. [104] prepared a 3D self-supporting CNF framework, modified with bimetallic oxide TiNbO_4_ (CNF/nc-TNO), via electrospinning and heat treatment. Ti and Nb atomic vacancies generated by the carbon thermal reaction introduced negative charges, regulating the local electric field and chemical environment around the CNF host, promoting the uniform migration and deposition of Li^+^. DFT in Figure 5a demonstrated that the bimetallic oxides containing Ti and Nb were more lipophilic to CNF hosts compared to single metal oxides. Therefore, the LMBs exhibited an ultra-long cycle life of over 4000 h (Figure 5b). Yang et al. [105] obtained a (MgZnO/CNF) triple-gradient scaffold by anchoring the CNF matrix with bimetallic compound MgZnO nanoparticles. The superhydrophilicity of MgZnO nanoparticles enhanced the wettability of CNFs and promoted the rapid infiltration of molten lithium. The triple-gradient, large specific surface area and good electronic conductivity promoted uniform lithium deposition on the composite anode, and effectively alleviated the growth of lithium dendrites. Therefore, the modified anode achieved a long cycle life and small overpotential even at a high current density of 50 mA cm^−2^ and a high area capacity of 10 mAh cm^−2^.

Complex Metal Compound/CNFs Composites

In order to more effectively protect the lithium anode, some researchers have introduced more than one type of metal compound into the CNFs matrix. Zeng and his co-workers [106] constructed a hierarchical porous hollow CNF composite scaffold decorated with nano ZnS and micro MoS_2_ (MS-ZS@PHC). Porous scaffolds limited the volume expansion of the anode during the cycling process, and the multi-scale lipophilic phases regulated the Li^+^ nucleus to make it uniformly grow on the scaffold (Figure 5c). Based on MS-ZS@PHC, the symmetrical battery exhibited a uniform lithium plating/stripping behavior. Different metal compounds have different lipophilic properties, and introducing them at different positions within the matrix can form a lipophilic gradient. Based on this principle, Liu et al. [107] embedded ZnO and Co_3_O_4_ into CNFs to form a dual gradient anode host (ZnO-Co_3_O_4_/CNF) (Figure 5d). ZnO and Co_3_O_4_ with different lipophilic properties formed a lipophilic gradient and a Li nucleation overpotential gradient, thereby promoting uniform lithium deposition. Moreover, the CNFs with highly conductive network buffered the stress from volume changes. Therefore, the full battery exhibited excellent electrochemical performance.

In recent years, heterostructure materials have shown potential advantages in the field of electrochemistry [108]. Heterogeneous materials are composed of heterogeneous regions with significantly different mechanical or physical properties, and the interaction and coupling between these heterogeneous regions can produce synergistic effects, making them possess superior comprehensive properties than homogeneous materials [109,110]. Taking advantage of this properties, Wei et al. [58] designed a ZnF_2_/ZnS heterostructure and N-doped carbon (NC) co-modified PCNFs (ZnF_2_/ZnS/NC/PCNFs) via the MOF-assisted method and carbonization processes. Figure 5e,f confirm the existence of a heterogeneous structure based on ZnF_2_ and ZnS. Then, the functional interlayer was coated on the commercial PP separator to stabilize the lithium anode. On one hand, CNFs with a large specific surface area and high conductivity network reduced the local current density and improved the interface compatibility between the anode and the interlayer. On the other hand, the heterogeneous interfaces accelerated charge transfer kinetics and significantly enhanced redox reactions. As expected, the modified battery exhibited the lowest charge transfer resistance of 2.8 Ω, excellent redox reaction kinetics and cycle stability (Figure 5g).

#### 3.2.4. Inorganic Non-Metal Material/CNFs Composites

**Figure 6:**
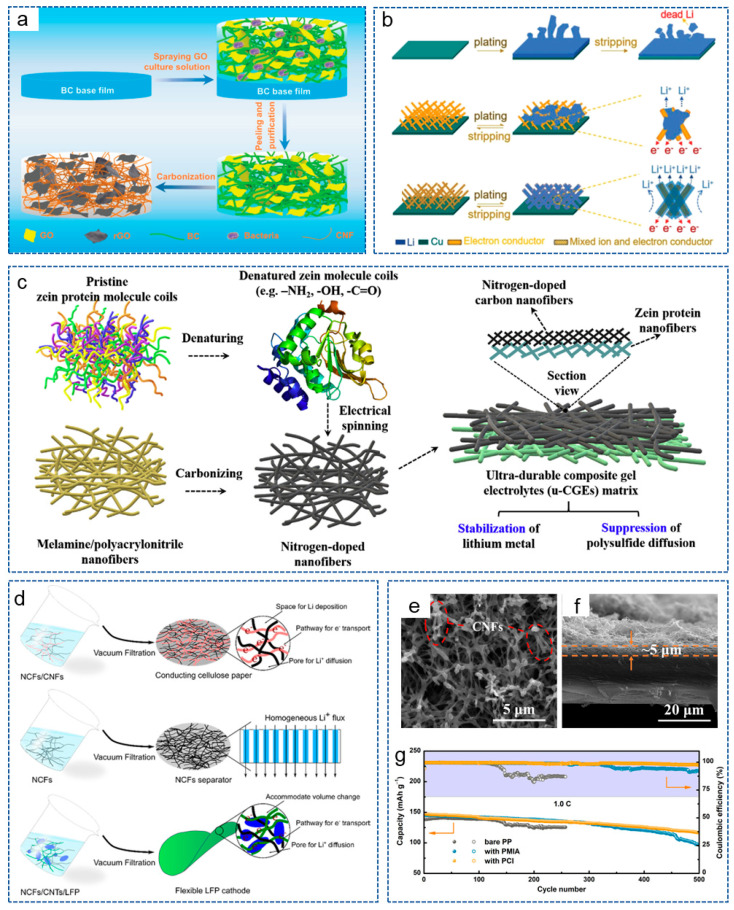
(**a**) Schematic illustration of the preparation process for BC-CNF@rGO [113]. Copyright 2022, Wiley-VCH. (**b**) Schematic illustration of lithium plating/stripping behavior on different electrode [114]. Copyright 2018, Wiley-VCH. (**c**) Schematic illustration of fabrication process for u-CGE [115]. Copyright 2020, Elsevier. (**d**) Schematic illustration of the preparation process for NCFs-based separator and electrodes [116]. Copyright 2018, American Chemical Society. (**e**) Top-view and (**f**) cross-sectional SEM images of PP with PCI. (**g**) Cycling performance of cells at 1 C [117]. Copyright 2020, Elsevier.

We all know that non-polar carbon materials have poor lipophilic properties, which prevents them from uniformly depositing Li^+^. As mentioned earlier, in order to increase the lipophilic properties of carbon materials, some lipophilic heteroatoms, metals and metal compounds have been introduced into the carbon matrix and have achieved satisfactory results. However, some cumbersome material preparation processes make it unsuitable for mass production and large-scale applications. Therefore, some inorganic non-metallic materials (such as CNTs, graphene oxide, etc.) with outstanding lipophilic property and conductivity, have also been used to regulate the lithium deposition behavior. For example, Wu et al. [111] obtained CNT/CNF scaffolds using the electrospinning method, providing sufficient space for lithium deposition. The introduction of CNTs effectively increased the conductivity, lipophilic properties and specific surface area of the system, thereby promoting electron transfer and reducing the local current density. In addition, reduced graphene oxide (rGO) also exhibits excellent lipophilic properties due to its abundant oxygen-containing functional groups on the surface [112]. Xu et al. [113] constructed a host for LMA using the in situ biological manufacturing method: CNF derived from bacterial cellulose@rGO nanosheets (BC-CNF@rGO) in a composite bracket (Figure 6a). The rGO provided abundant lipophilic functional groups, which coordinated with the conductive 3D network, effectively regulated the deposition behavior of lithium and promoted the reaction kinetics.

With the advancement of research, the concept of mixed ion and electron conductor (MIEC) has been proposed. Luo et al. [114] obtained an MIEC network by incorporating ion-conducting Li_6.4_La_3_Lr_2_Al_0.2_O_12_ (LLZO) nanoparticles into electron-conducting 3D CNF scaffolds. As shown in Figure 6b, compared with Cu foil and 3D electron conductors, the CNF skeleton of MIEC reduced the current density. In addition, the LLZO nanoparticles compensated for the depletion of Li^+^ during plating, thus greatly promoting the diffusion and distribution of charges, achieving ultra-high volume and area capacity, as well as rate capacity.

#### 3.2.5. Polymer/CNFs Composites

In addition to inorganic materials, some organic polymers, including biomass-based materials, have enormous application potential in the field of LMBs due to their rich polar functional groups, excellent mechanical properties or unique pore structures [118,119], etc. Yang et al. [115] fabricated an ultra-durable composite gel electrolyte (u-CGE). As shown in Figure 6c, by sample electrospinning, denatured zein protein molecules were deposited on the N-doped CNFs to form a 3D asymmetric matrix. Due to the numerous polar groups (-OH, NH_2_, -COOH), zein protein nanofibers skeleton demonstrated excellent lyophilic properties and ionic conductivity. Thanks to the dual action of pyrrole/pyridine N in N-CNFs and the polar groups of zein protein nanofibers, the assembled battery achieved uniform lithium deposition behavior and a dendrite-free anode. Nyholm et al. [116] also realized the effective combination of biomass-based materials with CNFs. They obtained a 3D porous conducting cellulose-paper (CCP) current collector composed of nanocellulose fibers (NCFs) and CNFs through ultrasound and heat treatment. Meanwhile, the NCFs-based separator and electrodes were also obtained (Figure 6d). The addition of NCF could serve as a flexible component to help form self-supporting 3D porous CCP with a large surface area and sufficient space. Therefore, the LMBs adapted well to the volume changes during the charging and discharging process and presented an outstanding cycling stability of 85% capacity retention after 1000 cycles at 2 C. Based on the excellent film-forming and mechanical properties of PMIA, Wang et al. [117] prepared a CNF/PMIA porous conductive interlayer (PCI) using the phase transformation method and coated it on the commercial PP separator (Figure 6e,f). During the cycling process, an equipotential surface was created on the lithium metal/PCI interface, thus eliminating the tip effect and unifying the Li^+^ flux. As a result, the Li|LFP battery exhibited a capacity retention rate of 78.9% after 500 cycles at 1 C (Figure 6g).

#### 3.2.6. Co-Doping/CNFs Composites

**Figure 7:**
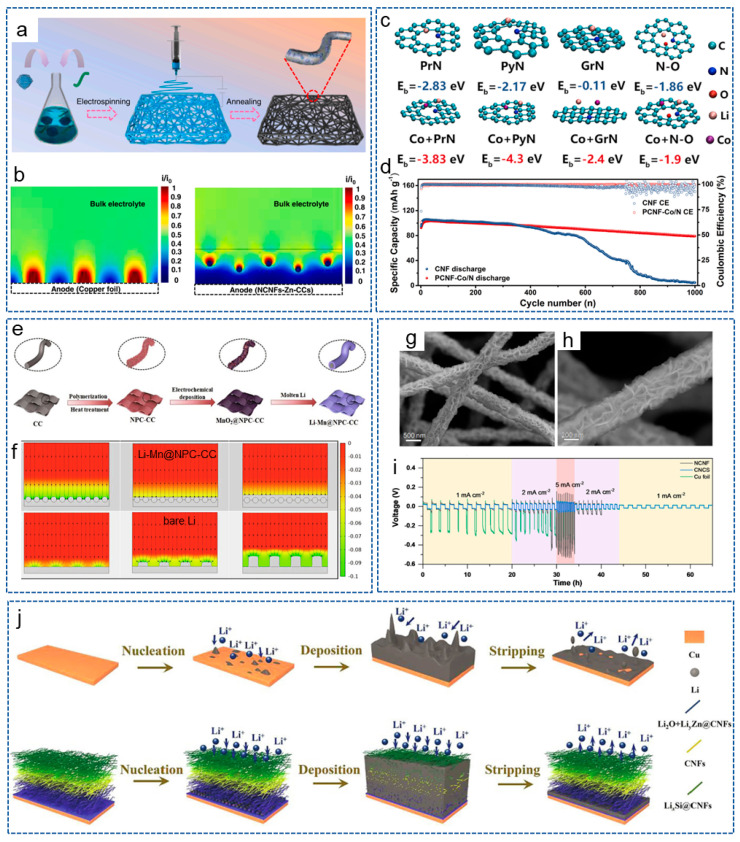
(**a**) Schematic diagram of the synthesis process for NCNFs-Zn-CCs. (**b**) Schematic diagram of the relative intensity distribution of localized electric field on NCNFs-Zn-CCs and Cu foil [120]. Copyright 2023, Elsevier. (**c**) Interaction of lithium atoms with PrN, PyN, GrN and N-O in CNF and PCNF-Co/N. (**d**) Cycling performance of PCNF-Co/N@LiǀǀLFP cells at 5 C [122]. Copyright 2021, Elsevier. (**e**) Schematic diagram of Li-Mn@NPC-CC fabrication process. (**f**) Simulation images of Li^+^ flux distribution on bare Li and Li-Mn@NPC-CC electrodes at deposition times of 0, 2000 and 4000 s via finite element method [124]. Copyright 2022, Elsevier. (**g**,**h**) SEM images of CNCS. (**i**) Rate performance of different symmetrical cells [125]. Copyright 2022, Wiley-VCH. (**j**) Lithium platting/stripping behavior on Cu and SCZ [126]. Copyright 2021, Elsevier.

Some studies have fully utilized the advantages of metals, metal compounds or heteroatoms, etc., and combined them with CNFs through co-doping to fully leverage their respective advantages and achieve the effective regulation of lithium deposition behavior. The combination of metals or metal compounds with heteroatoms can exert a synergistic effect, jointly serving as nucleation sites for lithium to reduce overpotential and guide the uniform lithium deposition. Therefore, various studies are dedicated to developing CNFs through the co-doping of metals or metal compounds with heteroatoms. Duan et al. [120] designed a Zn–carbon hybrid nanocage-embedded self-supporting N-doped 3D porous CNF interlayer (NCNFs-Zn-CCs) to inhibit the growth of lithium dendrites (Figure 7a). As shown in Figure 7b, the uniformly distributed Zn nanoparticles and N-doped carbon as the nucleation sites effectively reduced the nucleation overpotential. The 3D CNFs skeleton provided sufficient space for lithium deposition while also eliminating charge aggregation. Wu et al. [121] prepared a 3D porous and self-supporting scaffold for LMA composed of Co-decorated N-doped CNTs grown in situ on continuous CNFs by electrospinning. Analogously, Liu et al. [122] constructed a Co/N co-doping PCNF (PCNF-Co/N) via pyrolysis of self-assembled Co/Zn-MOF nanosheets on CNF. As shown in Figure 7c, the synergistic effect between Co and N defects transferred graphitic N into lithiophilic sites and improved the lithium affinity of pyrrolic and pyridinic N. These regular nucleation sites induced homogeneous lithium plating/stripping. As a result, the PCNF-Co/N@Li composite anode exhibited a long cycle life (Figure 7d).

Zeng et al. [123] prepared 3D porous hollow CNFs based on MOF-derived N/ZnO co-doped carbon frameworks and embedded CNTs (MC@HCNFs). Due to the decomposition of MOF nanoparticles at high temperatures, the spherical cavities were formed inside the fibers and served as a host for lithium nucleation and deposition. Meanwhile, the rational N/ZnO heteroatoms regulated the preferential deposition of lithium metal and inhibited the growth of lithium dendrites. Based on the MC@HCNFs, the symmetrical battery showed a ultralong lifespan of 2000 h with a low overpotential at 1 mA cm^−2^. Wang and his coworkers [124] designed MnO_2_ nanosheet-modified N, P co-doping CNFs on carbon cloth (MnO_2_@NPC-CC) via electrodeposition technology, and then obtained a composite anode (Li-Mn@NPC-CC) through the molten-infusion lithium strategy (Figure 7e). The doping of lipophilic heteroatoms and Mn-based compounds significantly enhanced the infiltration of molten lithium on the surface of CNFs and promoted the formation of a stable SEI. In addition, the porous and stable carbon framework ensured the rapid transfer of ions/electrons and improved the structural integrity during material preparation and battery cycling. Therefore, the framework achieved homogenization of the Li^+^ concentration and the corresponding current density, resulting in uniform lithium deposition on the electrode (Figure 7f). As a result, the Li-Mn@NPC-CC composite anode could stably cycle for 2200 h with a voltage hysteresis of 20 mV at 1 mA cm^−2^. Moreover, Li et al. [125] obtained a 3D host by coating 2D ultrathin NiCo_2_S_4_ nanosheets onto the self-supporting 3D interlaced N-doped CNFs (CNCS) (Figure 7g,h). The unique host exhibited high lipophilic properties and an extremely low lithium diffusion rate, which effectively confined the lithium metal in the gaps of 2D NiCo_2_S_4_ nanosheets through physical and chemical dual effects, thereby achieving uniform lithium deposition and preventing the formation of lithium dendrites. As expected, the Li/CNCS symmetric battery displayed a long lifespan and outstanding rate performance (Figure 7i). In addition, the Li|LFP battery also exhibited superior performance.

In order to guide the homogeneous bottom-growth of LMA, Peng et al. [126] prepared a double-gradient lithiophilic 3D Si@CNFs@ZnO-ZnO-Cu (SCZ) skeleton by electrospinning and magnetron sputtering technology and acted as the current collector of LMBs. Compared with Cu, the conductivity and overpotential gradient driven from the top LixSi@CNFs and bottom LiyZnO@CNFs effectively avoided the “top-growth” of lithium metal and achieved the uniform bottom-growth (Figure 7j). Therefore, the modified symmetric battery displayed excellent performance of 900 h at 1 mA cm^−2^. Furthermore, Pei et al. [127] constructed a porous Cu/Cu_3_P-N-CNFs 3D lithiophilic host with a high specific surface area for LMA, which reduced the local current density and alleviated the volume expansion during the charging and discharging process. In addition, the Cu/Cu_3_P heterostructure and N-doped carbon sites greatly reduced the nucleation overpotential. Therefore, the Cu/Cu_3_P-N-CNFs electrode exhibited a high coulombic efficiency of 94% for 500 cycles under 1 mA cm^−2^ and 1 mAh cm^−2^.

In conclusion, The CNFs-based composites obtained by combining a carbon matrix with lipophilic heteroatoms, metals, metal compounds, polymers, inorganic non-metallic materials, etc. greatly enhance the interaction between CNFs and lithium. These substances are uniformly dispersed in CNFs and can serve as the nucleation sites to guide the uniform deposition behavior of lithium. However, this treatment may cause defects in CNFs, leading to reduced conductivity, mechanical stability, and poor chemical stability. At the same time, the synergistic mechanism between various substances during the co-doping process has not been well explained. Therefore, it is necessary to explore the complex interactions and/or trade-offs between different key performance parameters.

## 4. Conclusions and Perspectives

Lithium metal is considered a potential ideal electrode material for the development of high energy density batteries. However, the presence of lithium dendrites hinders the commercial application of LMBs. Carbon nanofiber materials possess the advantages of high conductivity, large specific surface area and designable structure, making them a promising strategy for constructing a dendrite-free anode. In this article, we systematically reviewed several common preparation methods of carbon nanofiber materials and compared their respective characteristics. In addition, the research progress on CNFs in protecting LMA and improving the electrochemical performance of LMBs in recent years was summarized. Although significant achievements have been made, there are still some problems and challenges to be faced. The following are some of the main challenges that carbon nanofiber materials face in constructing dendrite-free LMAs.

First, LMBs are complex systems involving multiple interfaces. Currently, most of the reported methods for inhibiting the growth of lithium dendrites using carbon nanofiber materials are through simple comparative experiments to explore the role of CNFs. In fact, the introduction of CNFs will bring about changes in multiple interfaces and system composition. The pathway of how Li^+^ participates in electrochemical reactions and the mechanism of action of CNFs are still unclear. Thus, advanced characterization techniques, especially in situ characterization, as well as advanced theoretical simulations and single-fiber energy storage devices are needed to systematically study the structural evolution and interface evolution mechanisms during lithium plating/stripping processes, and deeply explore the electron/ion transport pathways and rates in CNFs.

Second, among several common preparation methods for CNFs, electrospinning technology is the most widely applied. At present, there is still great development space in the large-scale production of CNFs for stabilizing LMA. In addition, the production cost of CNFs with special structures (such as grading, branching and core-shell) is relatively high, and there is still a certain distance from industrial application. In the future, in order to achieve the large-scale preparation of carbon nanofiber materials, on the one hand, it is urgent to improve the process flow and equipment structure of the existing preparation technologies, and on the other hand, it is necessary to develop new preparation technologies for CNFs.

Third, the precise regulation and optimization of CNFs is of great significance for a more efficient and stable LMA. On the one hand, the structure, size, mechanical properties and other parameters of the CNFs matrix directly affect the deposition behavior of lithium. The introduction of CNFs inevitably results in additional weight and volume, which in turn affects the energy density of the entire battery system. Although flexible CNFs can theoretically improve mass energy density and simplify the preparation processes, obtaining lightweight and flexible CNFs with both high mechanical strength and low thickness is not an easy task. On the other hand, in order to enhance the interaction between non-polar carbon materials and lithium, researchers usually introduce lipophilic materials such as heteroatoms, metals, metal compounds, and biomass-based materials into the carbon matrix. However, the uniformity of these materials is difficult to accurately guarantee, and this treatment may cause defects in CNFs, leading to decreased conductivity and mechanical stability, as well as poor chemical stability. Therefore, it is necessary to systematically explore the complex interactions and/or trade-offs between different key performance parameters to achieve optimal performance.

In summary, CNFs and their composite materials have broad application prospects in lithium anodes due to their high conductivity, 3D network structure, flexibility, porosity and tunable properties, etc. At present, research on CNFs-based materials has effectively promoted uniform and reversible lithium plating/stripping behavior, significantly improving the electrochemical performance of LMBs. It is believed that carbon nanofiber materials will play a greater role in the development process of lithium anodes with the rapid development of new technologies and continuous accumulation of experience.

## Figures and Tables

**Table 1:** Comparison of various techniques for producing CNFs.

Preparation Technology	Diameter	Length	Electrical Conductivity	Cost
CVD	50–200 nm	50–100 µm	10^3^–10^4^ S cm^−1^	>500 USD/kg
Templated method	50–200 nm	1–60 µm	1–10 S cm^−1^	N/A
Electrospinning	10 nm–10 μm	10^3^–10^4^ µm	1–600 S cm^−1^	N/A
